# Comparison of a new transcutaneous bilirubinometer (Bilimed^®^) with serum bilirubin measurements in preterm and full-term infants

**DOI:** 10.1186/1471-2431-9-70

**Published:** 2009-11-12

**Authors:** Tanja Karen, Hans Ulrich Bucher, Jean-Claude Fauchère

**Affiliations:** 1 Clinic of Neonatology, University Hospital Zurich, Frauenklinikstrasse 10, CH - 8091 Zurich, Switzerland; 2 Department of Neonatology, University Children's Hospital Tuebingen, Calwerstrasse 7, D - 72076 Tuebingen, Germany

## Abstract

**Background:**

The gold standard to assess hyperbilirubinemia in neonates remains the serum bilirubin measurement. Unfortunately, this is invasive, painful, and costly. Bilimed^®^, a new transcutaneous bilirubinometer, suggests more accuracy compared to the existing non-invasive bilirubinometers because of its new technology. It furthermore takes into account different skin colours. No contact with the skin is needed during measurement, no additional material costs occur. Our aim was to assess the agreement between the Bilimed^® ^and serum bilirubin in preterm and term infants of different skin colours.

**Methods:**

The transcutaneous bilirubin measurements were performed on the infant's sternum and serum bilirubin was determined simultaneously. The agreement between both methods was assessed by Pearson's correlation and by Bland-Altman analysis.

**Results:**

A total of 117 measurement cycles were performed in 99 term infants (group1), further 47 measurements in 38 preterm infants born between 34 - 36 6/7 gestational weeks (group 2), and finally 21 measurements in 13 preterm infants born between 28 - 33 6/7 gestational weeks (group 3). The mean deviation and variability (+/- 2SD) of the transcutaneous from serum bilirubin were: -14 (+/- 144) μmol/l; -0.82 (+/- 8.4) mg/dl in group 1, +16 (+/- 91) μmol/l;+0.93(+/- 5.3) mg/dl in group 2 and -8 (+/- 76) μmol/l; -0.47 (+/- 4.4) mg/dl in group 3. These limits of agreement are too wide to be acceptable in a clinical setting. Moreover, there was to be a trend towards less good agreement with increasing bilirubin values.

**Conclusion:**

Despite its new technology the Bilimed^® ^has no advantages, and more specifically no better agreement not only in term and near-term Caucasian infants, but also in non-Caucasian and more premature infants.

## Background

The incidence of neonatal hyperbilirubinemia has been reported to be between 30-60% in full-term newborns and nearly 100% in premature infants [1]. The accurate measurement of bilirubin concentrations is essential for the diagnosis of hyperbilirubinemia and for guiding the clinician with regard to treatment. Although flawed by some imprecision, the gold standard remains the measurement of serum bilirubin concentration. This method however is invasive, painful and costly in terms of workload, time and money. Moreover, repeated blood samplings may lead to significant blood loss, which may be of particular concern in preterm infants. Trying to overcome these drawbacks, non-invasive methods of bilirubin measurements have been proposed. Transcutaneous bilirubinometry has been shown to correlate with serum bilirubin concentration in term infants [2-8]. In preterm infants transcutaneous bilirubinometry is less accurate than in term infants, as results are affected by the immature skin and by a different albumin-to-bilirubin binding [6,9,10].

Race, gestational age (GA), and body weight are other factors that may decrease the accuracy of the transcutaneous bilirubin (TcB) measurements. Most importantly, the most widely used transcutaneous devices tend to underestimate bilirubin values, particularly at higher concentrations [11,12].

Recently, a new 2^nd ^generation transcutaneous bilirubinometer (Bilimed^®^) has been developed, and based on a new technology using a five-wavelength analysis, a higher measurement accuracy has been suggested, independently of gestational age and skin colour. Only one study has so far been published with this new device in white Caucasian infants born near term or at term [13]. The aim of our study was to determine the accuracy and agreement of this new transcutaneous device with serum bilirubin concentration in newborn infants of different gestational ages and different skin colour.

## Methods

This study was carried out in the maternity ward and in the neonatal intensive care unit of the University Hospital Zurich. We intended to include 100 healthy term (maternity ward) and 50 preterm infants born between 28 0/7 and 36 6/7 weeks of gestation (maternity ward, intermediate care and NICU). We also aimed enrolling infants with different skin colours (Caucasian and non-caucasian origin). No infant had been treated with phototherapy until enrolment. The study was approved by the local ethics committee, and parental consent was obtained.

The Bilimed^® ^(Nufer Medical, Guemligen/Berne, Switzerland) is a microprocessor controlled device with 10 LEDs (3 green, 3 yellow, 2 blue, 2 red) which do not move during measurement and which is based on a five-wavelength analysis. Based on a minimum of two TcB readings, this device calculates the patient's bilirubin concentration and retains the best result. The bilirubin concentration is clearly displayed on the touch screen in μmol/l or mg/dl. It takes into account different ethnic skin colours, it does not necessarily need contact with the skin for measurement, and it generates no additional material costs for each measurement. In order to keep the measurement distance between the LEDs and the skin constant, a soft ring provided by the manufacturer was used, and the Bilimed^® ^was applied on the sternum of the infants. For our study, the transcutaneous bilirubin concentration was measured three times. This was done by the same person and immediately before or within 15 minutes after capillary blood sampling for serum bilirubin concentration measurements. The latter determinations were performed in the clinical chemistry laboratory of the University Hospital Zurich (Diazo method, Total Bilirubin Special COBAS Integra Neonatal, Roche Diagnostics, Basle, Switzerland). For our clinical chemistry, the variation coefficient for neonatal capillary bilirubin concentration measurements amounts to 4.6%.

For analysis, the mean of the three transcutaneous bilirubin readings was taken and then compared with the serum bilirubin concentration. The coefficient of variation (CV) of these three measurements was calculated for the three groups. The relationship between transcutaneous and serum bilirubin values was determined using simple linear regression analysis. The strength of this relationship was quantified by using the Pearson correlation coefficient r. The agreement between both methods was assessed by the method of Bland-Altman. A multiple linear regression analysis was performed to identify confounders to predict serum bilirubin. Excel (Microsoft Office Professional 2003, SP3) and StatView (Version 5.01 for Windows, SAS Inc., Carry, NC, USA) were used for statistical calculations and graphs. Statistical significance was defined for p < 0.05, with a maximum risk of α-error set at 5%.

## Results

A total of 99 term and 51 preterm infants were included in the study, out of whom 90 were of Caucasian origin and 60 were non-Caucasian (36 of Hispanic or Middle-East, 9 of African and 15 of Asian origin). The group of preterm infants was subdivided into more mature premature infants born between 34 0/7 and 36 6/7 weeks gestational age (group 2) and the more premature infants born between 28 0/7 and 33 6/7 weeks gestation (group 3). 111 measurements were performed in 99 term infants (group 1), 47 measurements in 38 preterm infants of group 2 and 21 measurements in 13 preterm infants allocated to group 3. The patient characteristics are shown in Table 1.

**Table 1:** Patient characteristics of the study group (TSB = total serum bilirubin)

	Group 1 (n = 99)		Group 2 (n = 37)		Group 3 (n = 13)	
	median	range	median	range	median	range
Gestation (wk)	39.1	37-42.3	36	34.1-36.5	30.3	29-32.2
Birth weight (g)	3300	2510-4950	2362.5	1570-3020	1360	1160-1790
Postnatal age(d)	4	2-12	4	2-7	5	2-11
TSB (μmol/l) (mg/dl)	223 13	35-349 2-8.4	181 10.6	95-262 5.6-15.3	195 11.4	81-224 4.7-13.1
Bilimed (μmol/l) (mg/dl)	198 11.6	50-343 2.9-20	202 11.8	99-264 5.8-15.4	195 11.4	76-256 4.4-14.9

**Figure 1:**
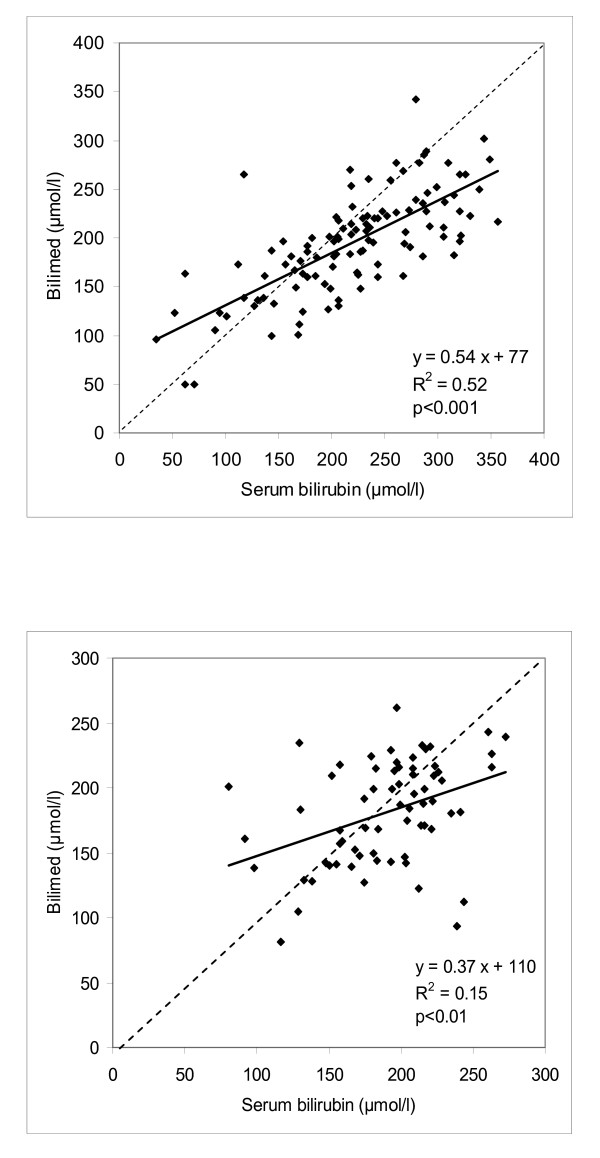
**Bilimed^® ^values on the sternum against total serum bilirubin values in term infants (Figure 1A, group 1, n = 111) and in preterm infants (Figure 1B, group 2 and 3, n = 68)**. Continuous linear regression line; dotted line of identity.

**Figure 2:**
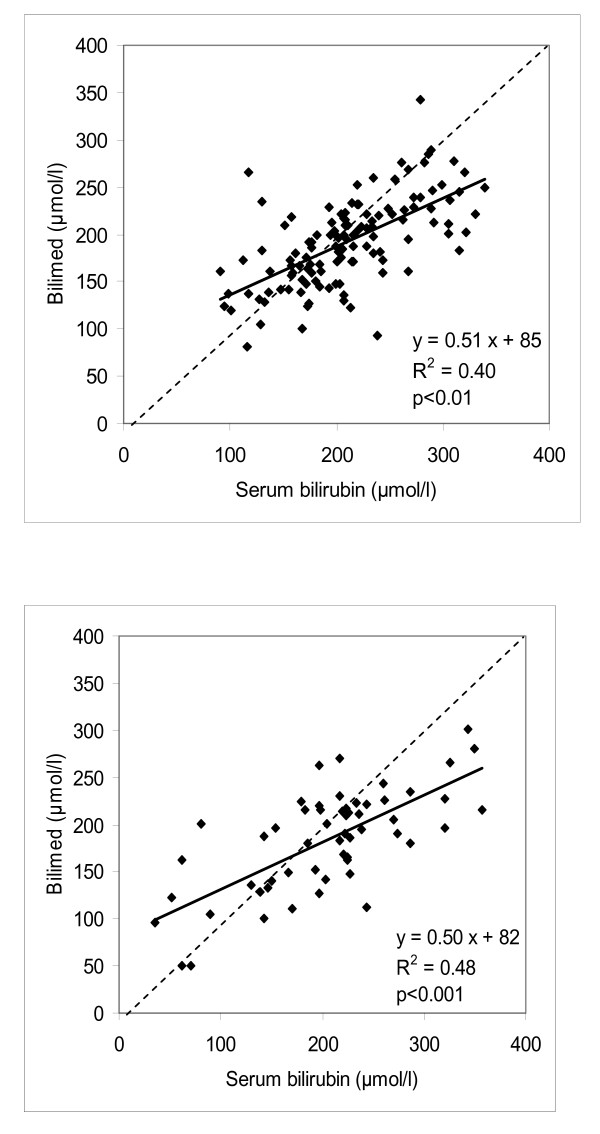
**Bilimed^® ^values on the sternum against total serum bilirubin values in Caucasian (Figure 2A, n = 126) and non-caucasian (Figure 2B, n = 59)**. Continuous linear regression line; dotted line of identity.

The relationship between serum bilirubin concentration and transcutaneous bilirubin concentration for the 3 gestational age groups was y = 0.54x + 75 for group 1, y = 0.38x + 127 for group 2, and y = 0.15x + 61 for group 3. Figure 1A depicts this relationship for all term infants (y = 111; y = 0.54x+77, R^2 ^= 0.52; p > 0.01), and Figure 1B for all preterm infants (group 2 and 3; y = 68; y = 0.37x + 110; R^2 ^= 0.48; p < 0.001). The same relationship is shown for all Caucasian infants and for all non-Caucasian infants in Figure 2A (n = 126; y = 0.51x+85; R^2 ^= 0.40; p < 0.01), and Figure 2B (n = 59; y = 0.50x+82; R^2 ^= 0.48; p < 0.001) respectively.

The Pearson's correlation coefficients r for the three groups were 0.722 (p < 0.001) for the group 1; 0.370 (p = 0.01) for group 2; and 0.521 (p = 0.016) for group 3. The variability of the three readings from a single site given infant is assessed by the coefficient of variation, which was 0.063 for group 1, 0.061 for group 2, and 0.062 for group 3.

**Figure 3:**
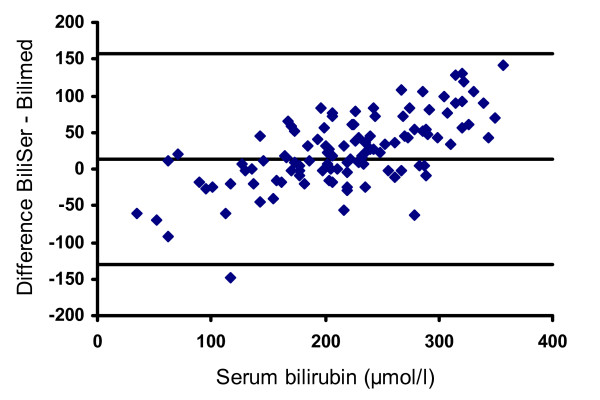
**Bland-Altman plot showing agreement between total serum bilirubin values and the Bilimed^® ^values for group 1**. The lines represent mean difference (-14) and ± 2SD of the difference (+/- 144)

**Figure 4:**
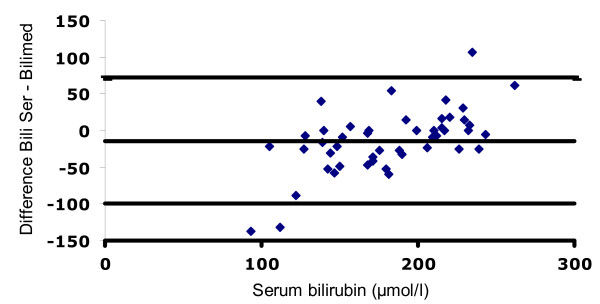
**Bland-Altman plot showing agreement between total serum bilirubin values and the Bilimed^® ^values for group 2**. The lines represent mean difference (+16) and ± 2SD of the difference (+/- 91)

**Figure 5:**
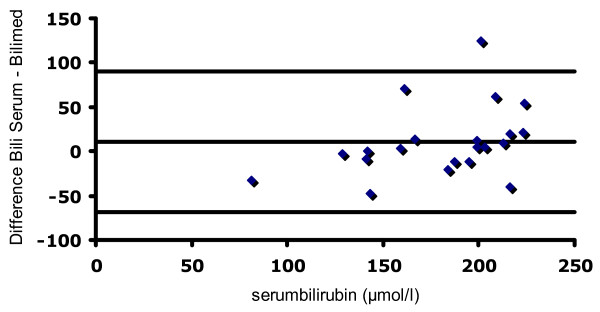
**Bland-Altman plot showing agreement between total serum bilirubin values and the Bilimed^® ^values for group 3**. The lines represent mean difference (-8) and ± 2SD of the difference (+/- 76)

**Figure 6:**
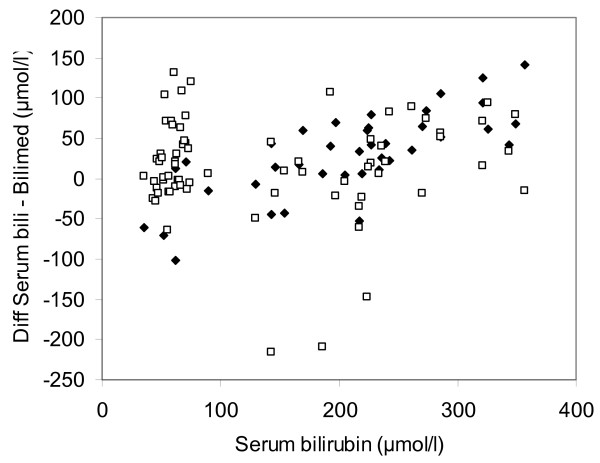
**Bland-Altman plot showing agreement between total serum bilirubin values and the Bilimed^® ^values for Caucasian (white rectangles, dotted lines indicate mean (16) and ± 2SD of the difference (+/- 121) and for non-caucasian (black diamonds, continuous lines represent mean (10) and ± 2SD of the difference (+/- 174)**.

A Bland-Altman plot of all comparisons is shown in Figures 3, 4 and 5. The mean deviation and variability (+/- 2SD) between the transcutaneous values and serum bilirubin in group 1 was: -14 (+/- 144) μmol/l (Figure 3), +16 (+/- 91) μmol/l in group 2 (Figure 4), -8 (+/- 76) μmol/l in group 3 (Figure 5). Figure 6 depicts the agreement between TcB and total serum bilirubin for Caucasian infants (white rectangles, mean deviation 16; variability +/- 121), and for non-Caucasian infants (black diamonds, mean deviation variability +/- 174).

A multiple stepwise linear regression analysis to predict serum bilirubin revealed birth weight (t-value 3.4, p < 0.001) as a confounding factor in addition to Bilimed values (t-value 10.7, p < 0.001), but not skin colour (t-value -0.05, p = 0.38), nor gestational age (t-value -0.05, p < 0.96).

## Discussion

Assessing a new 2^nd ^generation transcutaneous bilirubinometer Bilimed^®^, our study shows that the correlation and the agreement between serum and transcutaneous bilirubin measurements with the Bilimed^® ^does not confer any advantage over other transcutaneous devices. On the contrary, this device performs slightly worse when compared to the BiliCheck^® ^or to the Minolta-JM 103^® ^[2,8,10,13-16]. Recently, De Luca et al. (2008) found that in comparison to Bilimed^®^, there was a better correlation between BiliCheck^® ^and total serum bilirubin. This group performed their measurements only in white Caucasian full-term or near-term infants with a gestational age >34 weeks [13]. Our study adds to the available knowledge in that it provides also data on the performance of the Bilimed^® ^in non-Caucasian, and also in younger preterm infants.

### Term infants

In full-term infants, a good correlation between transcutaneous and conventionally determined serum bilirubin values was shown in different studies [12,16-19]. Other authors concluded, that TcB measurements cannot accurately predict serum bilirubin and that most of the transcutaneous devices have limitations at higher bilirubin values [8,11,12,20]. Beck and co-workers, investigating the BiliCheck^®^, pointed out, that the limits of agreement were too wide, and these authors concluded that TcB measurement can only be recommended as a screening tool [20]. Similary, the calculated limits of agreements are too wide to be acceptable in a neonatal clinical setting in order to use the Bilimed^® ^instead of Bilirubin measurements. Moreover, and as seen in Figure 3 the observed trend towards a worse agreement with increasing bilirubin values is of clinical concern.

### Preterm infants

Several studies have been performed in preterm infants, mainly enrolling healthy preterm babies. Our data do not confirm previous findings of a strong positive linear relationship between TcB measurements and plasma bilirubin concentrations in preterm infants [4,9,21-24]. The preterm infants in our study were divided into two groups with a cut-off at 34 0/7 weeks. The reason for sub-dividing this group was that the most very premature infants clinically clearly represented a separate group by being sicker or needing support (eg. parenteral feeding, coffein, oxygen, etc.), and thereby, in addition to the younger GA per se, were at higher risk for bilirubin encephalopathy. In both sub-groups, the relationship between transcutaneous and serum bilirubin was less strong in comparison to the studies stated above. Comparing the regression equation of all preterm infants (Figure 1B) with the term group (Figure 1A), the constant for the preterm infants was greater than for term infants (110 vs. 77). The correlation coefficient R^2 ^(0.15) and the Pearson's coefficient r (0.39) showed a worse agreement than for term infants (R^2 ^= 0.52, and r = 0.72 respectively). In comparison, De Luca et al. found a R^2^-value of 0.20 and r-value of 0.45 for the whole group of term and near-term infants [13].

Several confounding factors have been described previously, such as gestational age, postnatal age or skin condition [6,9,21,23,25]. Karolyi et al. demonstrated that only TcB measurements and birth weight were independently associated with the plasma bilirubin concentration in a group of very low birth weight (VLBW) infants <1500 g [22]. Gestational age, postnatal age, intrauterine growth retardation (IUGR) and hematocrit did not turn out to be of significant importance. Knüpfer et al. reported correlation coefficients ranging from 0.47-0.67 in the age group of infants born at 23-30 weeks [9].

Importantly, we observed many difficulties with the measurements in the group 3. Factors accounting for these measurement difficulties were smaller body size with a narrow thorax area as a sampling site, and the fact that many of these very preterm infants were studied while still having a respiratory distress syndrome with retractions and tachypnoea. The rapidly and extensively moving sampling site made transcutaneous measurements cumbersome and even virtually impossible in some of this tiny infants. If feasible at all, the time needed for tcB measurement was two or three times longer to get a reading of acceptable quality. Obviously, the advantage of the Bilimed^® ^device of not needing to touch the infant's skin did not overcome these problems. Therefore we stopped the transcutaneous measurements in this gestational age group after 13 patients with, as a consequence, a substantional higher number of more mature preterm infants (group 2) within a total number of 51 preterm infants studied.

### Different skin colours

Importantly, for both Caucasian and non-Caucasian infants, the constant in the regression equation was far different from zero, but interestingly, they were not different from each other. Moreover, both the Pearson's correlation coefficient r and R^2 ^were similar for both groups (Figure 2A and 2B). When the agreement between both groups was assessed by Bland-Altman analysis, there were a few more TSB measures outside the limits of agreement in the Caucasian infants, but the limits of agreement were much wider in the non-Causasian infants (+/- 174 vs. +/- 121) (Figure 6). In order to asses the influence of gestational age, birth weight and ethnicity as confounding factors, we performed a multiple linear regression analysis which showed that birth weight is a confounding factors but not skin colour, nor gestational age.

The accuracy of TcB readings may also differ when comparing different ethnic groups and the hope that TcB measurements would be independent of both race and pigmentation has not been fulfilled yet [5,8,9,17]. In one study by Bhutani and co-workers the correlation between BiliCheck^® ^TcB and high performance liquid chromatography (HPLC) measurements of the total serum bilirubin (TSB) in African-American infants was as good as in Caucasian infants, whereas TcB measurements by the Minolta JM-103 tended to overestimate the TSB measurements in African-American [2,16]. In the other ethnic groups however (East Asian, Middle Eastern, Indian/Pakistani, Hispanic), Minolta JM-103^® ^measurements were similar to the TSB measurements [26,27]. Slusher et al showed a good agreement between BiliCheck^® ^and TSB in indigenous African infants [28]. In our study the agreement between serum bilirubin values and Bilimed^® ^in non-white infants (Hispanic, Middle East, African and Asian) was not affected by skin colour when compared with white infants.

### Performance, site of sampling and serum bilirubin measurement

As it has been shown that the accuracy of the measurements decreases when performed by several investigators, we took care that all measurements were performed by one person solely, and always over the infant's sternum [11,14,29]. Choosing the sternum as sampling site is due to the large measurement area of the Bilimed^® ^instrument making measurement over the forehead more difficult, especially when studying more premature infants due to their smaller size or to nasal CPAP bonnet. Maisels et al., using the JM-103 Jaundice Meter, performed measurements over the forehead and sternum in 475 infants and found a higher correlation for the values assessed over the sternum [16]. Because the forehead is exposed to ambient light, this can lead to underestimation of TSB values, while the sternum is nearly always covered [5,30]. Measurements over the sternum was therefore considered the better choice for our measurements in our study. For the purpose of this study, we determined serum bilirubin concentrations in our central hospital lab. Although we use our own assay on the NICU (ABL 835 Flex, Radiometer, Copenhagen, Denmark) for the daily routine with the advantage of being faster, less labor-intensive and less expensive, the central laboratory remains our 'gold standard' as it regulary participates in multi-centre quality assessments. In order to minimize errors in the pre-analytical phase, the samples were shielded against light and high-speed internal pneumatic delivery system allowed for prompt measurement.

## Conclusion

Our results show that, despite its new technology the new transcutaneous bilirubinometer Bilimed^® ^has no clinical advantages, and more specifically no better agreement with the serum bilirubin compared with the existing non invasive bilirubinometers. It remains to be shown in studies including a greater number of infants of different ethnic background with different skin colours if the Bilimed^® ^performs better in these situations when compared in a head-to-head testing to other transcutaneous devices.

## Abbreviations

GA: Gestational age; HPLC: High performance liquid chromatography; IUGR: Intrauterine growth retardation; NICU: Neonatal intensive care unit; TcB: Transcutaneous bilirubin; TSB: Total serum bilirubin; VLBW: Very low birth weight infants

## Competing interests

The authors declare that they have no competing interests.

## Authors' contributions

TK had primary the responsibility for the protocol development, patient screening, enrollment, data analysis and writing the manuscript. HUB contributed to the writing of the manuscript, and to the revision JCF was responsible for the design of the study, supervised the execution of the study and data analysis, contributed to the writing of the manuscript and to the revision. All authors read and approved the final manuscript.

## Pre-publication history

The pre-publication history for this paper can be accessed here:

http://www.biomedcentral.com/1471-2431/9/70/prepub
